# A scoping review of the use of visual methods in supporting women with substance use histories

**DOI:** 10.1057/s41599-026-07293-x

**Published:** 2026-04-22

**Authors:** Raginie Duara, Netalie Shloim, Anna Madill

**Affiliations:** University of Leeds, Leeds, UK

**Keywords:** Health humanities, Psychology

## Abstract

Problematic use of substances is a growing global issue, however only 1 in 18 women with this issue seek treatment and interventions have been critiqued for overlooking gender-specific needs. Given the rich history of visual methods in research and therapy, our aim is to conduct a scoping review exploring how visual methods are used in research involving cisgender women with a substance use history, with a particular focus on their potential to support recovery. Visual methods, in this review, refers to approaches that use images or other visual creations, such as digital storytelling, photovoice, drawing, collage, and visual elicitation techniques. These methods offer non-verbal modes of expression that can support narrative agency and help tackle stigma, trauma, and relational complexities often experienced by women in recovery. Five databases, Medline, PsychINFO, Embase, Web of Science, and SCOPUS were searched to identify peer-reviewed empirical papers that address women’s recovery from substance use and employed visual methods where women participated in the visual creations. Of 5776 initial records, 27 articles were identified. Using content and thematic analysis, we outline how visual methods are used in these articles and what we can learn about the potential of visual methods in supporting women’s recovery. Articles were divided into two groups. The first have direct relevance to recovery and consist of three themes capturing process of change: Gaining temporal context, Transformation beyond reflection, and Power of shared stories. The second have indirect relevance to recovery and consist of two themes identifying pathways to recovery: Informing services and Developing community support. In conclusions, visual methods have immense potential in supporting women’s recovery through making their experiences tangible and as a tool for knowledge translation bridging the gap between research, practice and policy to inform gender-responsive support systems.

## Introduction

Globally, between 2012 and 2022, there has been a 20 percent increase in drug abuse with estimates suggesting the involvement of 292 million people (United Nations Office on Drug and Crime, 2024). Problematic use of substance is a growing concern with patterns of use associated with harm, impaired functioning, or psychosocial consequences consistent with descriptions commonly used in public health and clinical research (American Psychological Association, 2024; Cleveland Clinic, 2024; Canadian Public Health Association, 2024). Approximately 64 million people suffer substance use disorder (SUD), of which one in seven men and only one in eighteen women seek treatment (United Nations Office on Drug and Crime, 2024). Recovery in the context of substance use is complex, fragile, and gendered (Covington, 2008). As a demographic, women with SUD are vulnerable to a range of interpersonal and systemic abuses which impact also their children, if they are a mother. It is essential to find tools that are conducive to understanding and supporting the specific needs of women in their recovery journey (White, 2007).

This scoping review explores how visual methods are used in research involving cisgender women with substance use histories including tobacco use, our particular focus the potential of such methods to support recovery. Visual methods have been shown to provide exceptional means for expression and reflection in the context of research into SUD (e.g., Duara et al., 2022). Hence, we identify and synthesise the contemporary research in this field to provide an overview of the visual methods used, how they have been used, and to speculate on the potential for their development for supporting women in recovery. This also meant that recovery was not used as an inclusion criterion for this review. Rather recovery functioned as an analytic focus through which we explore the potential application of visual methods. Hence, consistent with scoping review methodology, we refrained from imposing a recovery framework and relied on each study’s conceptualisation of substance use and recovery where present. However, we recognise that recovery is not just about abstinence from substance use but involves the development of a new identity (Laudet, 2007). This includes physical health, personal growth, and social reintegration (Best et al., 2012). Gender is implicated throughout (Grella et al., 2008) since identity change is shaped by gendered biological, social, and psychological factors, as outlined below.

Women have physiological characteristics making them more susceptible than men to the biological damage of drug abuse. For instance, differences in body fat, hormonal makeup and metabolism may impact how substances are processed and consequently vulnerability to effects (Becker and Koob, 2016; Greenfield et al., 2010). This can shape how women experience embodiment and potential futures, in turn influencing sense of self in their recovery journey. Furthermore, women experience more pronounced social taboos with regard to substance use than do men which can exacerbate guilt and shame impacting, not just how women see themselves and their place in the social world but causing barriers to seeking help (Cloud and Granfield, 2008). Finally, psychological distress and interpersonal problems are more prevalent among women drug users (Grella et al., 2008) influencing identity construct and potentially leading to poorer mental health and greater support needs even beyond active use (Andresson et al., 2021). Noteworthily, such differences in impact and experiences around addiction is even more pronounced among the transgender community (Connolly et al., 2025) meaning that they face unique challenges including transphobia, discrimination, and barriers to gender-affirming care (Greaves, 2020). These complexities call for a dedicated analysis beyond the scope of the current review.

Traditional recovery models have made significant progress in addressing substance use. They have, however, been critiqued for their ‘one-size-fits-all’ approach which can overlook the gender-specific relational dynamics, structural barriers, and societal stigmas that women encounter (Covington, 2008). For example, women commonly initiate substance use while in intimate relationships, often posing as a means of coping with abuse or coercion (Covington and Surrey, 2008). The relational model of women’s psychological development suggests that healing and self-expression are often rooted in developing a safe, caring, authentic connection as opposed to traditional approaches of growth towards self-sufficiency and independence (Covington and Surrey, 2008). Merely group reflections guided by shared images, for instance, can potentially help deepen solidarity and build a sense of community and meaningful relationship (Agner et al., 2023). Moreover, structural barriers experienced most acutely by women, such as fear of losing child custody and lack of childcare, can impact open dialogue, especially when women feel morally judged (Shrimpton et al., 2024). This can lead women to internalise shame and silence (ibid). In this context, visual methods such as photovoice and drawing, offer tangible ways of capturing the “interplay of action, meaning and context” (Rhodes and Fitzgerald, 2006, p. 360) through externalising complex emotions not easily conveyed through words (Manley et al., 2015). Other possible mechanism include surfacing embodied experience (Malchiodi, 2020), enhancing agency (Smith et al., 2024), and providing a safe space for meaning-making that does not rely solely on verbal articulation (Smith et al., 2024; Malchiodi, 2020).

Visual methods are not only methodologically innovative but also ethically responsive to the lived realities of women in recovery. However, recovery services tend to emphasise verbal or written communication as the primary tool in assessment and intervention. On the other hand, visual methods are relatively well-established in clinical practice, such as in art therapy and expressive arts interventions (Junge, 2015; Malchiodi, 2020). Images have been integral to therapeutic practices for over a century (Junge, 2015). Carl Jung is often regarded as the originator of art therapy in the early 20th century with regard to the painting dream imagery. Other early pioneers included Margaret Naumburg and Edith Kramer who posited that art could be a conduit for accessing and processing unconscious thoughts and emotions, offering an alternative to traditional talk therapies (Tobin, 2015). However, art therapy as a concept is usually assigned to Adrian Hill, a professional artist, who started to paint as a kind of therapy for himself and in 1942 coined the term ‘art therapy’ (Bitonte and Santo, 2014).

Art therapy evolved to incorporate various visual media such as drawing, painting, and sculpture to address a wide range of psychological issues including trauma, anxiety, and depression (Moon, 2010). The non-verbal nature of these methods may be particularly beneficial for those who found it challenging to articulate their experiences verbally (Bitonte and Santo, 2014). For instance, digital storytelling (Mazzoli Smith et al., 2024) and therapeutic photography (Loewenthal, 2020) emerged as contemporary therapeutic practices allowing clients to create and share visual narratives of their troubling experiences. The process may facilitate externalisation and, through this, create a minimal distance to catalyse insight and healing (Malchiodi, 2020; Milasan et al., 2022).

Concurrently, visual research methods also have a rich history that dates back to the late 19th century (Bailey and McAtee, 2003). Photography was one of the initial visual methods to gain traction, at first for its ability to illustrating an objective reality, and later for its value in depicting subjective perspectives (Prosser and Loxley, 2008). As technology advanced, the scope of visual methods expanded (Reavey and Brown, 2021). For example, film was used as a tool to document in anthropology from the 1940s (Prosser and Loxley, 2008). Visual methods became popular in psychological research more recently. At first visual methods were used to record what is observable and later to support the predominantly oral or written, meanings of research participants, possibly because the polysemous nature of visual material makes it complex to use as data in itself (Reavey and Brown, 2021).

The digital revolution of the late 20th and early 21st centuries further transformed visual research methods (Vivienne and Burgess, 2013). Accessibility of digital cameras and, later, smartphones democratised the production of visual data, enabling both researchers and participants to capture and share images easily. This facilitated innovative participatory methods, such as photovoice, where participants use photography to document and discuss their experiences (e.g., Chonody et al., 2013; Snow-Hill et al., 2024), which has been utilised also as an advocacy tool in disciplines such as public health (Holm, 2008; Wang and Burris, 1997). New technologies gave rise to methods such as digital storytelling where participants created narratives accompanied by video that provided a voiceover, photographs, and sometimes music (Vivienne and Burgess, 2013). These kinds of new visual methodologies are particularly effective in research with women because they amplify voices that are often marginalised and help challenge dominant narratives by representing lived experiences through images and storytelling (Azzarito, 2023). These approaches also support participant agency and empowerment in self-representation much less mediated by the researcher than previously was the case (Mazzoli Smith et al., 2024), allowing women to reclaim narrative power and engage in critical reflection, potentially leading to social change and greater self-advocacy (Nitia, 2024).

In summary, visual methods have transitioned from rudimentary documentation tools to transformative modes of communication in both research and therapeutic contexts. Their evolution reflects a growing appreciation of the multifaceted ways in which visual media can enhance understanding, expression, and recovery (Duara et al., 2022; Stuebing et al., 2020; Reynolds and Lim, 2007). However, the use of visual methods in research on women with substance use histories remain fragmented making it difficult to understand how they have been used and to speculate on the potential for their development for supporting women in recovery. Hence, we have undertaken a scoping review which aims to explore how visual methods are used in research involving cisgender women with substance use histories, with a particular focus on their potential to support recovery efforts.

## Method

To provide a clear overview of how studies were selected, we summarise the eligibility criteria below describing each in detail.

### Inclusion criteria

1. Gender-Specific Target Population: Studies focusing on cisgender women to ensure gender-specific insights. Where included studies described participants using sex terms (“male/female”), we retained this language in order to accurately represent each study’s reporting, but our interpretation focuses on gendered experience rather than biological sex.
2. Substance Use History: Studies where the target population consists of individuals with a documented history of substance use (e.g., drug or alcohol dependence, misuse, or addiction) including tobacco given its addictive properties (World Health Organisation, 2023) and often co-occurring with other substance use, particularly among women (Prochaska et al., 2004). We did not define ‘substance use history’ ourselves but relied on each study’s own criteria provided that the study identified participants as having some form of past or present substance use relevant to recovery. This ensures that the study population is relevant to the exploration of substance addiction and recovery.
3. Publication Date Range: Published within the last 10 years (2014–2024) to ensure the research is current and reflects contemporary contexts and challenges.
4. Language: Studies published in English to ensure the ability to interpret the findings without language barriers.
5. Visual Methods: Studies that utilise visual methods such as photo-elicitation, video ethnography, participatory visual research, or any method involving visual data.
6. Participant-Produced Visual Materials: Include studies where the visual materials (e.g., photographs, videos, artwork) are produced by the participants as part of the research, ensuring authentic engagement with participants.

### Exclusion criteria

1. Mixed-Gender Studies: Studies that address multiple genders without providing separate data or analysis for cisgender women, as this may dilute the gender-specific findings relevant to our research question.
2. Transgender Population: Studies focusing on transgender populations, as this population warrants unique factors beyond the scope of our study.
3. No Substance Use History: Studies where the target population has no history of substance use or where substance use is not clearly defined or reported, as these studies would not contribute to understanding the role of visual methods in addiction and recovery.
4. Non-Empirical Studies:

- Opinion pieces, editorials, and non-peer-reviewed articles
- Grey literature (e.g., government reports, theses, or dissertations)

5. Substance Use Not Primary Focus:

- Studies where substance use is not a primary focus, even if it is mentioned.
- Studies primarily focused on other issues, such as mental health or criminal behaviour, without a significant focus on substance use.

6. Incomplete Data on Gender: Studies that do not provide distinct or separate reporting of outcomes specifically for cisgender women, making it unclear how this population is impacted or represented.
7. Non-Visual Methods: Studies that do not use visual methods such as photo-elicitation, video ethnography, or participatory visual research, as the focus is on exploring risk and resilience through visual approaches.
8. Performative and Literary Arts-Based Methods: Studies primarily using non-visual arts-based methods such as theatre, dance, music, poetic enquiry, or narrative writing, unless visual data (e.g., video recordings, images) are a core component of the analysis.
9. Hybrid Methods with Non-Visual Focus: Studies that employ hybrid arts-based methods (e.g., combining visual, auditory, and performative elements) where the primary method is non-visual (e.g., performance or sound) and visual data are secondary or peripheral.
10. Researcher-Produced Visual Materials: Studies where the visual materials are created by the researchers (e.g., documentary films or illustrations made by the research team), as participant-produced visuals are a key focus.
11. Use of Online Visual Materials without Participant Engagement: Exclude studies that rely solely on visual materials from online sources (e.g., YouTube videos, Instagram posts) without direct engagement with participants, as the focus is on participant-driven visual production.

#### Search strategy

The authors worked together to create categories of search words that were sufficiently broad, ensuring relevant articles are not lost, and finally included three categories–substance use, women and visual methods. Keywords around these three categories were refined for each database, adapting to optimise search sensitivity. For example, controlled vocabulary terms were adapted where MEDLINE used MeSH terms such as exp substance addiction/, EMBASE used Emtree terms like exp drug addiction/, and PsycINFO applied APA Thesaurus terms such as exp drug abuse/. Additionally, participatory research terms varied, with MEDLINE using exp participatory research/ and PsycINFO using exp participatory action research/. Peer-reviewed empirical studies were searched in five databases in September 2024 that included Medline, PsychINFO, Embase, Web of Science, and SCOPUS.

#### Screening and selection of articles

**Fig. 1:**
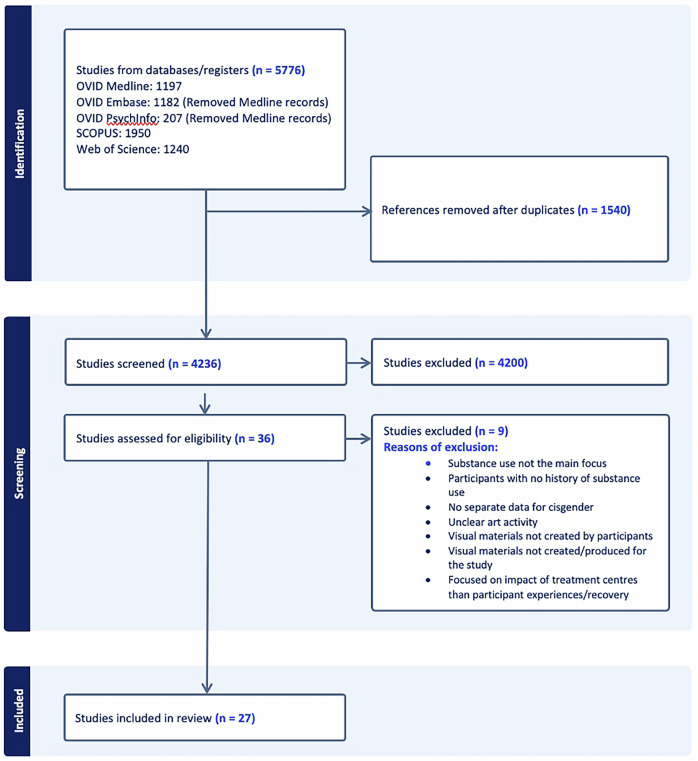
PRISMA chart.

A total of 5776 articles were initially imported to Rayyan, a review software, and after removing duplicates, we were left with 4236 articles. The first author reviewed the titles and abstracts of these articles, with (*blinded*) and (*blinded*) independently double-checking 5% of the articles each to ensure rigour and accuracy, i.e., 10% of the total. For a few articles, relevance could not be determined only through title and abstract screening which required checking full text. No discrepancies appeared at this stage between reviewers. After the initial screening, full text of 36 articles were reviewed by the first author and where there were doubts (*n* = 4), (*blinded*) and (*blinded*) reviewed for decision to include. Discrepancies were resolved along with the first author. Nine articles were excluded from the 36 articles for reasons including, substance use not being the primary focus, participants with no history of substance use, no separate data between cisgender and transgender, unclear art activity, visual materials not created by the participants and focus on impact of treatment centres than on participant’s experiences/recovery. The final included articles (*n* = 27) were checked for relevance by all three authors. Figure 1 shows the PRISMA chart of the selection process.

#### Data extraction

The data extraction sheet included the following records: general information (title, author/s, year), study characteristics (purpose, population, substance studied, methodology), data collection (method of data collection apart from visual, type of visual method, purpose of the visual method), type of analysis, outcomes related to SUD (general findings, gender-specific insight, cultural relevance), potential of visual methods as explicated by the articles’ authors (therapeutic benefit, challenges of using visual methods for recovery), and practical implications/recommendations associated with the methods by the respective authors, if any.

#### Analytical procedure

A combination of **content analysis** and **thematic analysis** was employed to synthesise the data extracted from the included studies. This dual approach allowed for both the quantification of visual methods used in recovery-related interventions and the exploration of underlying mechanisms associated with their impact on women’s recovery journeys.

**Fig. 2:**
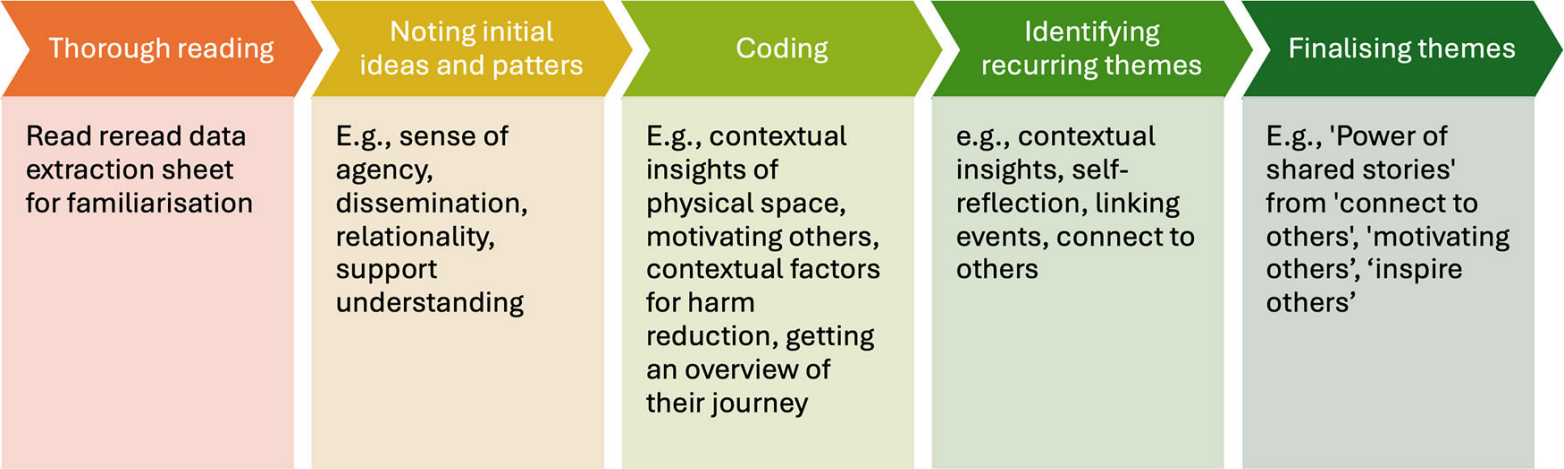
Steps of thematic analysis of the data extraction sheet.

Content Analysis was conducted to identify and categorise the types of visual methods (e.g., photography, drawing, digital storytelling) used across the 27 included studies. The frequency and distribution of these methods were recorded to map trends and highlight areas of focus within existing research. Simultaneously, thematic analysis (following Braun and Clarke, 2006) was applied to examine how visual methods contribute to recovery. As a first step, the data extraction sheet was read and reread to gain familiarity and in-depth understanding, noting initial ideas and patterns of visual methods usage in all 27 studies. This was followed by coding and identifying recurring themes that reflected the mechanisms through which visual tools facilitated recovery. It was an iterative process between the three authors, with the first author initiating the first round of codes and themes which were subsequently refined through several discussions with the second and third authors. In this way, themes were developed iteratively through an inductive process, ensuring that patterns identified within the data were grounded in the experiences reported by researchers and participants across the studies. Figure 2 demonstrates the steps in this analysis.

## Results

### Overview of the selected studies

Of the 27 studies selected, the majority (*n* = 25) employed qualitative methods, with one using quantitative methods (i.e., Hudson et al., 2024), and one adopting a mixed-methods approach (i.e., Haines-Saah et al., 2015). Visual methods varied across the studies: 17 used photography, six drawing, three digital storytelling (DST), and one used a combination of drawing and photos. ‘Drawing’ was a particularly varied method and, for our purpose here, includes anything that involved participants using pens or other relevant implements to project on an external surface. The studies were conducted predominantly in high-income countries (term as per World Bank, 2025): USA (*n* = 14), Canada (*n* = 5), both USA and Canada (*n* = 1), Australia (*n* = 2), France (*n* = 1), Belgium (*n* = 1) and Mexico (*n* = 1), with two exceptions: Nepal (*n* = 1: Dhital et al., 2023) and India (*n* = 1: Madill et al., 2023).

In terms of gender, seven had a target population of multiple genders in which results regarding the women could be, at least in part, disambiguated for the purposes of this review. The rest (*n* = 20) focused on women. Specific sub-groups of women were of interest in thirteen studies: substance use during pregnancy or in the context of motherhood (*n* = 7 : Greene et al., 2023; Howard and Colvin, 2021; Paterno et al., 2018, 2019, 2020; Wall-Bassett et al., 2014, 2016); the homeless or formerly homeless^1^ (*n* = 3: Bassi et al., 2020; Chang, 2017; Oudshoorn et al., 2021); physical disability (*n* = 1: Cordova et al., 2015); sex workers (*n* = 1: Syvertsen et al., 2017); and post-incarcerated (*n* = 1: Flentroy et al., 2015). In most, participants were aged 18 years and older, while two did not specify participant age (Greene et al., 2023; Syvertsen et al., 2017). Eight studies did not specify the substances used by participants, two included participants with different substances of choice, while others focused on polysubstance use (*n* = 7), opioids (*n* = 4), alcohol (*n* = 3), tobacco (*n* = 2), and cannabis (*n* = 1) (Table 1).

**Table 1:** Descriptive overview of the selected 27 studies.

Methodology	Visual method	Country	Gender	Age	Substance
qualitative = 25 quantitative = 1 mixed = 1	photos = 17 drawing = 6 DST = 3 drawing & photos = 1	USA = 14 Canada = 5 USA/Canada = 1 Australia = 2 France = 1 Belgium = 1 Mexico = 1 Nepal = 1 India=1	women = 19 mix men & women = 8	18 yrs + =25 unspecified = 2	unspecified = 8 different substance of choice = 2 polysubstance = 7 opioids = 4 alcohol = 3 tobacco = 2 cannabis = 1

### Synthesis

An important over-arching pattern is that the studies divide into two distinct groups. Content analysis indicated nine papers that address directly how recovery was aided through the use of visual methods. The remaining 18 leverage the knowledge generated through the use visual materials to enhance recovery programmes and/or to drive social change through community support or policy impact. Accordingly, the synthesis takes as its starting point that the studies can be divided into Group 1 labelled ‘Direct relevance to recovery’ and Group 2 labelled ‘Indirect relevance to recovery.’ Group 1 is characterised by three themes: ‘Gaining temporal context,’ ‘Transformation beyond reflection,’ and ‘Power of shared stories’ (Table 2). Group 2 is characterised by two themes each with a number of subthemes: ‘Informing services’ and ‘Developing community support’ (Table 3). While most authors contribute only one article to the corpus, Wall-Bassett et al. (2014, 2016) contributed two articles based on the same data set, both to Group 2, and, of the three articles by Paterno and colleagues based on the same data set, two were allocated to Group 1 (Paterno et al., 2018, 2020) and one to Group 2 (Paterno et al., 2019).

**Table 2:** Visual method and visual technique associated with each theme constituting Group 1 ‘Direct relevance to recovery’.

Themes	Visual method	Specific visual technique [UI]
Gaining temporal context	drawing (*n* = 3)	Healing Me Timeline [10] Road Drawing technique [13] Episodic future thinking [15]
	DST (*n* = 1)	DST [18]
Transformation beyond reflection	drawing (*n* = 3)	Small Body Outline Drawing [6] Imagery [21] Healing Me Timeline [10]
	drawing and photo (*n* = 1)	Bible journaling [9]
Power of shared stories	photos (*n* = 1)	Photovoice adaptation and SNS [12^a^]
	DST (*n* = 2)	DST [18, 19]

^a^mixed gender study.

**Table 3:** Visual methods and visual techniques associated with each subtheme constituting Group 2 ‘Indirect relevance to recovery’.

Themes	Subthemes	Visual method	Specific visual technique [UI]
Informing services	Specific conditions	photos (*n* = 7)	Photographed walking interview [4] Photovoice [5^a^, 11, 14] Photo-elicitation [24, 26] Photo narrative method [17^a^]
		DST (*n* = 1)	DST workshop [20]
	Generic contextual insights	photos (*n* = 6)	Photo-elicitation [1^a^] Photovoice [7^a^, 8, 23] Photo-led interview [27^a^] Photovoice & photo-elicitation [25]
		drawing (*n* = 1)	Vidaview Life Story Board [3^a^]
Developing community support	Visual dialogues	photos (*n* = 2)	Photovoice [14, 16]
	Encouraging change	photos (*n* = 9)	Photovoice [5^a^, 7^a^, 8, 11, 14, 23] Photo-elicitation [2, 22^a^] Photo-led interview [27^a^]
		DST (*n* = 1)	DST workshop [20]
		drawing (*n* = 1)	Vidaview Life Story Board [3^a^]

^a^mixed gender studies.

The synthesis now explicates the themes/subthemes of each group separately and in detail. For clarity, each study has been given a unique identifier (UI) from 1 to 27. UIs are used in Tables 2 and 3 to indicate the specific visual technique used so that the extent to which any one study contributes to more than one theme/subtheme is visually indexed.

#### Group 1: Direct relevance to recovery

The nine studies constituting Group 1 employed visual methods directly to impact the recovery of participants and/or others like them who had contact with the generated visual materials. Table 2 summarises the number of studies using a particular visual method and specific visual technique (with study UI) associated with each of the three themes. All but one of these studies (i.e., Haines-Saah et al., 2015) focused entirely on women. The studies were fairly evenly distributed across the identified themes, with three or four studies contributing to each theme. Most studies tended to be quite focused and were able to be characterised within one theme only. The exceptions were Flentroy et al. (2015) which involved both ‘Gaining temporal context’ and ‘Transformation beyond reflection’ and Paterno et al. (2018) which involved both ‘Gaining temporal context’ and ‘Power of shared stories.’ While drawing is the most popular visual method, the theme ‘Power of shared stories’ is associated with photos and DST only.

##### Theme 1: Gaining temporal context

Four of the nine studies of direct relevance to recovery used visual methods, specifically drawing and DST, to help participants connect with their past, present, and future. Increased temporal awareness fostered a sense of self-worth, resilience, and hope.

Flentroy et al. (2015) [UI10] exemplifies this approach with post-incarcerated women through the Healing Me Timeline: an intervention designed to help participants map out significant life events. Using a blank sheet of paper and coloured markers, participants responded to facilitator-led questions about pivotal experiences such as their first encounter with substances. While initially prescriptive, the intervention became more exploratory and allowed participants to delve deeply into specific life events they had marked. Similarly, Hanes (2017) [UI13] employed drawing as a method to help participants reflect on their life journey, using roads as a metaphor. Participants used drawings to represent the paths they were on as well as their desired futures. The freedom to control what they revealed fostered agency and self-realisation through the externalisation and processing of significant experiences.

Hudson et al.’s (2024) [UI15] approach of episodic future thinking aimed at reducing delayed discounting which is a tendency prevalent in substance use where immediate gratification is prioritised over future rewards. By creating a visual representation of their envisioned future home, participants reported a reduction in delayed discounting and increased resistance to substance use. Finally, Paterno et al. (2018) [UI18] demonstrated the therapeutic potential of DST as an intervention tool. By creating digital narratives of their recovery journeys, participants reflected on their experience of substance use during pregnancy and connected it to their current work as peer mentors supporting pregnant women with SUD. Besides the sharing of experiences, this process ignited a sense of purpose and hope as participants saw how their past struggles contributed to their present achievements and capacity to help others.

##### Theme 2: Transformation beyond reflection

Visual methods can be transformative in enabling individuals to express and process difficult emotions. For instance, in Flentroy et al.’s study (2015) [UI10: see also Theme 1], participants were able to use the Healing Me Timeline technique to surface silenced traumas and to reflect meaningfully on the connection to high-risk behaviours. By externalising abstract emotions and complex life circumstances these became more accessible, interpretable, and fostered personal transformation.

Dansky’s (2022) [UI6] case study highlights the profound impact of the Small Body Outline Drawing tool. By using colour, symbols, and words in her artwork, a participant depicted the strong emotions she felt within her body and transformed them into an “image of hope” (p. 24). During the process, she conveyed deeply personal experiences and feelings she had not previously shared in treatment. Both the act of creation and the subsequent discussion of the artwork had significant therapeutic value in terms of self-worth, validation, and pride in her progress. Similarly, through a case study, Skeffington and Browne (2014) [UI21] demonstrated the power of expressing experiences through imagery. They report that, through the creative process, the client was able to identify changes that needs to be made in her life to maintain her abstinence from alcohol.

Dillon (2021) [UI9] evaluated the technique of bible journalling to support recovery by interviewing five women engaged in the process which included the use of drawing and photographs. For example, Dillon notes how one woman drew transparent butterflies symbolising freedom and transformation over bible text, butterflies which became larger and more dynamic towards the right, capturing her journey of personal growth and recovery.

##### Theme 3: Power of shared stories

While drawing is the dominant visual method in the first two themes, photos and DST are shown here to be powerful tools to create shared stories for motivating change in others and fostering opportunities for participants to develop supportive peer networks. Haines-Saah et al.’s study (2015) [UI12] is a typical example where young people shared personal stories about tobacco use and cessation through images on Facebook, and supporting each other in the online platform, to the extent that some smokers who took part reported a decision to quit. In Paterno et al.’s study (2018) [UI18: see also Theme 1], women with experience of perinatal SUD engaged in a DST workshop, sharing their stories and, through this process, establishing a deep connection. Screening the created digital stories, participants acknowledged the immense potential of these narratives to inspire and motivate others toward recovery. In a subsequent study, participants reported positive responses sharing their stories in a closed Facebook group (Paterno et al., 2020) [UI19].

#### Group 2: Indirect relevance to recovery

The 18 studies constituting Group 2 leveraged the knowledge generated through the use of visual materials to enhance recovery programmes and/or to drive social change through community support or policy impact. Table 3 summarises the number of studies using a particular visual method and specific visual technique (with study UI) associated with each subtheme of the two themes. Fifteen studies contributed to theme ‘Informing services’ and 12 studies to ‘Developing community support,’ However, half (*n* = 9) were able to be characterised across both themes. Photos are overwhelmingly the most popular visual method, drawing and DST associated with only one study each: Boucher et al. (2017) and Paterno et al. (2019) respectively.

##### Theme 1: Informing services

Fifteen of the 18 studies in Group 2 used the understandings gathered through visual methods to inform the work of rehabilitation services. The theme ‘Informing services’ has two subthemes: Specific conditions and Generic contextual insights.

##### Subtheme 1: Specific conditions

Eight studies contributed to understanding specific conditions that can inform services, specifically regarding homelessness, pregnancy/motherhood, and physical disability. In these, visual methods, predominantly photos, were critical in understanding unique challenges faced by particular demographics of women to help inform services.

Chang (2017) [UI4] walked with formerly homeless women through their environment photographing significant sites. These helped reveal risk and protective factors shaping their substance use and recovery experiences enabling targeted recommendations for service providers. Similarly, half of the participants in Oudshoorn et al.’s study (2021) [UI17] were homeless and, although a mixed gender group, a photo narrative method again revealed specific risks faced by female addicts such as of human trafficking.

Five studies produced information informing services with regard to pregnancy and/or motherhood (i.e., UI14: Howard and Colvin, 2021; UI11: Greene et al., 2023; UI20: Paterno et al., 2019; UI24&26: Wall-Bassett et al., 2014, 2016). For instance, in a photo-elicitation study, Wall-Bassett et al. (2016) [UI26] explored the food choices of recovering women. They found that, in addition to the directives set by the residential programme, their children’s wellbeing and preferences had a significant impact on the women’s eating patterns. The authors suggest this information may be useful in health promoting strategies for this demographic. The one study in this sub-theme that did not use photos - Paterno et al. (2019) [UI20] - employed DST to explore the experience of pregnancy during addiction and recovery and the participants’ current experiences as peer mentors for women in similar circumstances. For many, the responsibility of becoming a mother was a huge motivation to sustain recovery. In one digital story, the camera pans out from the joyful participant to her two happy children as she tells the story of the positive impact on them of her recovery.

Finally, Cordova et al. (2015) [UI5] used photographs in group discussions with Latinos, including Latina women, exploring substance use within the broader context of physical disabilities. The photos heightened participants’ awareness of ecodevelopmental influences on their recovery and illuminated shared challenges, such as the role of family dynamics in substance use. This focus on cultural and gendered nuances allowed the authors to underscore the need for family-based interventions for Latina women who reported facing both cultural and gender-based discrimination.

##### Subtheme 2: Generic contextual insights

Seven studies explored generic contextual insights that can influence recovery efforts for women again predominantly with the use of photos. For example, influence of place of care on therapeutic alliance (UI1: Bailly et al., 2018), women’s daily situations impacting recovery (UI23: Van Steenberghe et al., 2021), perception of community assets in supporting harm reduction (UI8: Dhital et al., 2023), and general social, cultural and health discourses influencing smoking and quitting (UI25: Triandafilidis et al., 2018).

Hence, in a mixed gender, photo-elicitation study (UI1: Bailly et al. 2018), photos brought by women revealed information about the associations the physical space of a therapy centre triggered for them. For instance, the colours, design and furniture gave participants the impression of a prison interrogation room rather than its purpose as a therapeutic space. At the same time, the geometrical structure of a statue outside the centre conveyed a positive sense of boundaries which some acknowledged they needed to stay in recovery. These findings illuminate how services need to give careful consideration to the impact their physical design can have on clients.

Eight women in their recovery journey were interviewed in a photovoice study by Van Steenberghe et al. (2021) [UI23] to explore barriers and facilitators to their recovery process. Photographs revealed several aspects of their experience, such as influence of body image in initiating substance use, the shame and stigma they faced, and the significance of support from others in aiding their recovery process. Similar experiences were also revealed in Madill et al.’s photo-led interview study (2023) [UI27] where women in Assam reported facing stigma even during their recovery leading the authors to highlight urgent need for female-focused rehabilitation facilities and policies.

In the exception to the use of photos, Boucher et al. (2017) [UI3] employed the Vidaview Life story Board, generally used in therapeutic settings, to help injection drug users communicate their perception of harm reduction strategies. This involves a board, markers and picture magnets to create a visual portrayal of their lived experiences. Although there was no evaluation of women’s experiences in particular, the study did reveal and reflect on the finding that women who inject substances may experience more stigma and discrimination than do men.

##### Theme 2: Developing community support

Twelve of the 18 studies in Group 2 shared the potential of visual materials as a bridge between participant’s personal experiences and the community in order to reduce stigma and/or inform policy change. The theme ‘Developing community support’ has two subthemes: Visual dialogues and Encouraging change.

##### Subtheme 1: Visual dialogues

Two studies used photos produced by the participants to engage directly with the community in, what we are terming, visual dialogues.

In Howard and Colvin’s (2021) [UI14] study, pregnant and postpartum women shared their experiences of desperation, lack of control, needs and hopes. For example, one photo showed the participant’s hand reaching out to her frowning little daughter, another a car depicting ‘pregnant and homeless.’ Participants shared their photos at a conference addressing a large group of parent attorneys who represent this demographic, in this way conveying their experiences and connecting with the larger community. Similarly, in Nair et al.’s study (2016) [UI16], photos became bridge between female nursing students and faculty members facilitating conversations about risks and protective factors related to alcohol use: a recognised issue in this student population. This led to faculty recommending peer mentorship and annual seminars on healthy coping.

##### Subtheme 2: Encouraging change

Eleven studies used a range of visual materials to inform researchers in the effort to develop community-led initiatives. By highlighting the nature and extent of discrimination, visuals can communicate evocative messages to encourage the community to play an active role in reducing stigma, challenging barriers, and fostering a supportive environment for women with problematic substance use.

The only drawing-based study in this subtheme revealed stigmatisation faced by women that, as one participant noted, led to feelings of worthlessness and, in turn, to lying in order to hide their addiction (UI3: Boucher et al., 2017). And, in the one study that used DST (UI20: Paterno et al., 2019), mothers revealed the stigma faced during pregnancy from healthcare providers but also from others in the addiction community. However, the majority of studies in this sub-theme used photos of participants’ perception of their environment to gather information about the social and structural changes needed to reduce risks and increase protective resources to support women into recovery. For example, in both Dell et al. (2022) [UI7] and Dhital et al. (2023) [UI8], participants shared photos depicting desirable public physical spaces, such as green areas and community art facilities, that can support recovery.

In a study of a hyper-scrutinised demographic, Syvertsen et al. (2017) [UI22] called for changes in drug policy after investigating the impact on drug injecting female sex workers and their long-term partners living around the US-Mexico border. Photo-elicitation interviews facilitated critical social analysis by showcasing the daily lives of these couples and their community as caring and supportive that contrasted the violence commonly depicted in the media. This was used to propose adoption of drug policies focused on structural factors that avoid harming this already vulnerable population.

In a photovoice study of another highly scrutinised demographic - pregnant and breastfeeding women who consume cannabis - Greene et al. (2023) [UI11] revealed the intersectional stigma experienced when such women also belong to specific racial groups, such as the Black community. The authors suggest that using the visual materials generated in such research to cocreate resources such as educational tools may make these more accessible and attractive to relevant demographics. This was enacted successfully by Madill et al. (2023) [UI27] in a photo-led interview, mixed gender, study of young people’s resilience to and recovery from substance use in Assam in which the visual materials generated in the research was used to create and integrate into educational material, policy recommendations, and an awareness-raising social media campaign.

## Discussion

This article presents a scoping review of the way in which visual methods are used in research involving women with a substance use history, our particular focus the potential of such methods to support recovery. Although our review centres on gendered aspects of recovery, we recognise that cis-women’s experiences may be influenced by both biological (sex-based) and social (gender-based) determinants. The evidence base we reviewed, however, seldom distinguishes these domains. As a result, our findings reflect the gendered narratives present in the literature rather than biological mechanisms, which fall outside the scope of this scoping review. Twenty-seven articles met the inclusion criteria and our synthesis identified two separate groups. The first group were of direct relevance to the recovery of participants, i.e., to the potential of visual methods in clinical practice, and consists of three themes: ‘Gaining temporal context,’ ‘Transformation beyond reflection,’ and ‘Power of shared stories.’ The second group were of indirect relevance to recovery and consists of two themes: ‘Informing services’ (‘specific conditions’ and ‘generic contextual insights’) and ‘Developing community support’ (‘visual dialogues’ and ‘encouraging change’). This second group is focused on the potential of visual methods in research to promote positive change at a social level. We now consider what our synthesis illuminates with regard to how visual methods are used in research involving women with a substance use history and what this might tell us about the potential of such methods to support their recovery.

To commence, several general observations can be made. First, the majority of studies employed qualitative methodology. This reflects the history of the development of visual methods (Prosser and Loxley, 2008) and indicates that quantitative research is only beginning to integrate the benefits of visual data and its analysis, at least in the field of addiction research. Second, only two of the articles were conducted in low to middle resource settings, i.e., Nepal and Mexico. This indicates broader geographical gap in the evidence and suggests scope for future research to examine how widely available digital devices, such as smartphones, might enable the use of visual methods in supporting women’s recovery in such contexts. Third, the majority were women-focused research exclusively. This suggests researchers often view women with a substance use history a distinct demographic with specific risks, challenges, and needs.

We now consider the three themes constituting group 1, i.e., those with direct relevance to recovery. Articles were almost evenly spread across the three themes suggesting that each is a key focus of interest, each label implying a potential *mechanism of change* promoted by the visual method(s) employed.

The first theme of group 1 is ‘Gaining temporal context.’ Three of the four articles in this theme used drawing to link past, present, and/or future events, facilitating a bird-eye view and, subsequently, supporting behaviour change. For example, Flentroy at al. (2015) noted that when women marked and discussed significant events in the ‘Healing Me Timeline’ activity, it offered a space to process the past and move into the future with more clarity. Of potential importance, it may be the *tangible* nature of visual representations that allowed the women to reflect on changes over time, facilitating insight into their progress which, in turn, empowered them to make informed decisions about their future. For some, this inculcated a sense of hope and gave their recovery purposeful direction because it was situated within a clear timeline.

The second theme of group 1 is ‘Transformation beyond reflection.’ All four articles in this theme used drawing to surface difficult memories and emotions in a supportive environment, fostering more positive mental health. For many, this act of expression marked a critical starting point for change through offering a new lens through which to initiate transformation (e.g., Dansky, 2022). However, a further mechanism of change was the way in which drawing allowed the women to represent their feeling also to others (e.g., Skeffington and Browne, 2014).

The third theme of group 1 is ‘Power of shared stories.’ Unlike the first two themes in this group, drawing was not the dominant visual method, rather the three articles used photos and DST. When visual representations through these media were created and discussed within groups, the process fostered a sense of relatedness. Sharing their recovery narrative with visual support engendered solidary and the materials became a source of motivation for others to consider their own path toward recovery.

To summarise, group 1 articles, i.e., those with direct relevance to recovery, utilised predominantly *drawing* and this appears to enhance *mechanisms of change* associated with surfacing, expressing and communicating of difficult events and emotions in tangible timeline which allows the past to be processed, a hopeful future to be envisaged, and relationships forged with others walking the same path.

It may be difficult to express in words the complex intersecting challenges that women drug users face and the emotional traumas leading to, and occurring as a result of, their drug use that need worked through in their recovery journey (Neale et al., 2014; Tuchman, 2010). Creating a *tangible* visual product is analogous to the first step of ‘scaffolding’ in narrative therapy in which the patient is able to name the problem (White and Morgan, 2006). This helps the patient move forward through no longer carrying the emotional burden of *being* the problem (Conner, 2017). A visual product can be symbolic mediator providing distance from the problem (Chan et al., 2012), such as reflecting on a timeline from the outside and as separate from self (e.g., Hanes, 2017; Flentroy et al., 2015). Suchman (2016) proposed that vulnerable groups have lower capacity for mentalisation. This is an *externalisation* process found helpful in populations with limited or damaged capacity for mentalisation. That is, to aid people make sense of their inner subjective world in relation to external reality (Daubney and Bateman, 2015). This may be very relevant to people with a drug use history because poor ability to mentalise is associated with reliance on external sources for self-regulation (Suchman, 2016). A person may tie their mental world too tightly to the external world leaving no space for alternative explanations. Conversely, if the mental world is too disconnected from the physical world, changes in external information will have little impact on internal processes (Daubney and Bateman, 2015).

Aligning with mechanisms of change posited in narrative and mentalisation-based therapy, visual methods may be of direct relevance to recovery by helping women slow down and reflect, providing an opportunity to bring the internal world and external reality into productive conversation. The emphasis on *drawing* in group 1 may facilitate this through being very intimate and personal, built-up over time and returned to, and within the reach of all.

We now consider the two themes constituting group 2, i.e., with indirect relevance to recovery. Articles in this group were reasonably evenly spread across the two themes suggesting, as with group 1, that each is a key focus of interest. Specifically, ‘Informing services’ and ‘Developing community support’ are two vital *pathways* for supporting women towards recovery promoted by the visual method(s) employed. Photography-oriented visual methods are the predominant approach in group 2 articles suggesting that they support both pathways well.

The first theme of group 2 is ‘Informing services.’ This consists about equally of two foci: ‘specific conditions’ and ‘generic contextual insights.’ In ‘specific conditions,’ challenges were revealed with regard to how intersecting identities influenced the recovery process. For example, photos by Latina women with physical disabilities revealed a need for family interventions (Cordova et al., 2015) and five articles focused on substance use in the context of pregnancy and motherhood. In ‘general contextual insights,’ it was revealed how issues such as the physical environment of the service can impact women’s recovery (Bailly et al., 2018), shedding light on the broader context in which these journeys unfold.

Hence, research using visual methods supports research indicating that women benefit particularly from women-only programmes (Ashley et al., 2003; Sun, 2006) in which their age (Chen et al., 2004), ethnicity (Marsh et al., 2021), employment (Haberle and White, 2007) and reproductive status, such as pregnancy (Chen et al., 2004), are taken into account. Moreover, the visually-informed research coheres with, and may facilitate insight into, the evidence that women often need trauma-informed residential treatment (Tompkins and Neale, 2018) and within service childcare facilities (Chen et al., 2004) so that they feel safe and supported to discuss physical and sexual abuse, sex work, and concerns related to children which may be associated with their substance use (Ashley et al., 2003; Sun, 2006).

The second theme of group 2 is ‘Developing community support.’ This consists of two foci: ‘visual dialogues’ and ‘encouraging change,’ with more studies falling into the latter category. In ‘visual dialogues,’ two articles used photos to invite the community into a supportive conversation to develop understanding about women in recovery (Howard and Colvin, 2021; Nair et al., 2016). In ‘encouraging change,’ visual methods, predominantly photos, revealed the detrimental impact of stigma and discrimination on women’s ability to engage in, and sustain, recovery (e.g., Greene et al., 2023). These articles provide insights informing community initiatives reducing stigma and supporting women in recovery.

More generally, group 2 articles are of indirect relevance to recovery via the process of *knowledge translation* informing two vital pathways for supporting women towards recovery: rehabilitation services and community support. Importantly, visual methods, particularly photos, allow complex experiences to be communicated in a direct, engaging, and relatable way to different audiences some of whom may not be, at least at first, sympathetic. In this way, the research has potential to be not only informative but transformative (Mertens, 2017), for example improving the way in which services are designed and implemented (Straus et al., 2009). Co-created films, posters and animations have been already shown to help communicate nuanced experiences and generate actionable evidence (Duara et al., 2022). By integrating visual methods into knowledge translation strategies, researchers and communities can work together to identify barriers, advocate for policy changes, and create more supportive environments for women’s recovery.

To our knowledge, although there are two systematic reviews that examine visual methods in mental health recovery (Jay et al., 2022; Milasan et al., 2022), this is the first review – systematic or scoping - that focuses specifically on addiction recovery, while addressing also gendered experiences within this context. We have integrated research from a variety of disciplines including psychology, public health, and gender studies to provide a comprehensive synthesis what this research tells us with a particular focus on the potential of visual methods to support recovery.

### Limitations

First, although we had a broad range of disciplines in our synthesis, we included only peer-reviewed articles so may have missed important grey literature, such as conference presentations which were not subsequently published. Second, the literature did not tend to include outcome information, so we have been unable to comment on the short- or long-term effectiveness or impact of visual methodologies with regard to direct or indirect relevance to women’s recovery. Third, the geographical and cultural acceptability and applicability of visual methods with women with a substance use history is largely untested and our restriction to English language papers may have meant that culture-specific perspectives in other languages was missed. Fourth, standard protocol often involves two independent reviewers screening for inclusion of all titles, abstracts and texts. However, due to resource limitations, we conducted an independent check on a sample of 10 percent. Finally, there is inconsistency in sex and gender terminology in the included studies which limits the extent to which biological (sex-based) and social (gender-based) influences on cis-women’s recovery can be disentangled. However, our review focused on the gendered narratives present in the literature rather than biological mechanisms which fall outside the remit of this scoping review.

### Future research

The potential of visual methods to support women in recovery is immense. Future work could explore if visual methods benefit other demographics in recovery in similar and/or different ways given indications of positive outcomes (e.g., Manley et al.’s, 2015, mixed gender study on the use of drawing and movement). For instance, future research is needed to explore how visual methods support recovery among transgender women, nonbinary people, and other gender-diverse populations. Only two studies in our corpus employed visual methods to promote a dialogue with the community. Hence, future research could explore further how visual narratives can be used in advocacy, policy reform, and public awareness campaigns to reshape societal attitudes toward women with a substance use history. Further work is needed also to understand how visual methods can supplement the recovery process, particularly in the context of poor literacy and language barriers. Moreover, the potential for using key visual methods such as drawing and photos in low resource settings, particularly low-and-middle-income countries, has hardly been touched. Finally, research is needed to evaluate the long-term impact of using visual methods in women’s recovery, improving services, and changing community attitudes. This might entail mixed methods research using quantitative outcome measures.

This scoping review of the way in which visual methods are used in research involving women with a substance use history indicates the value of these methods for capturing and supporting the recovery process of this demographic. This is facilitated directly because the visual methods employed (predominantly drawing) can support relevant *mechanism of change* and indirectly through knowledge translation (predominantly in the form of photos) informing vital *pathways* – services and community - supporting women towards recovery. Overall, this review encourages a greater focus on how visual methods can support recovery, shape services, and enhance community-based models tailored to women’s specific needs.

## Supplementary information

Supplementary information

Supplementary information

Supplementary information

## Data Availability

The materials supporting the findings of this review are available in the supplementary files accompanying this manuscript. These include: Data extraction and coding file: An Excel spreadsheet containing the full data-extraction matrix, initial codes, and thematic categories used in the analysis. Database export files: A ZIP archive containing the raw database export files from all searches.Search strategy documentation: A file detailing the full list of keywords, Boolean operators, and search strategies used across all databases.

## Footnotes

1 Terminology Note: Language used in this review reflects the terminology employed by the original studies. For example, while current literature often prefers terms such as “unhoused people,” some included studies used “homeless,” and this wording has been retained to accurately represent the source material.
