# Supplementary material for: A scoping review of the use of visual methods in supporting women with substance use histories
Source: Humanit Soc Sci Commun. 2026 Apr 22;13(1):964. doi: 10.1057/s41599-026-07293-x (PMC13314533; doi:10.1057/s41599-026-07293-x)
Supplement: Supplementary file 1 — Supplementary information [file 41599_2026_7293_MOESM1_ESM.pdf]

Aim: To explore how visual methods are used in research involving cisgender women with substance use histories including tobacco use, our particular focus the potential of such methods to support recovery

Keywords:

Female	Addiction	Visual methods
Woman<br>Women<br>Girl/s<br>Female/s	substance addiction<br>substance use<br>substance abuse<br>substance misuse<br>drug addiction<br>drug use<br>drug abuse<br>drug misuse<br>alcohol addiction<br>alcohol use<br>alcohol abuse<br>alcohol misuse<br>substance use disorder<br>drug dependence<br>alcohol dependence<br>substance dependence<br>opioid addiction<br>heroin addiction<br>cocaine addiction<br>cannabis use<br>marijuana addiction<br>alcohol dependence<br>nicotine addiction<br>tobacco use<br>stimulant abuse<br>hallucinogen use	Art/s<br>Arts-based method/s<br>Visual methods<br>Visual art<br>Participatory art<br>Participatory visual method<br>Creative expression<br>Creative engagement<br>Creative therapy<br>Creative process<br>Creative medicine<br>Printmaking<br>Silk screening<br>Expressive activity<br>Caricature*<br>Cartoon*<br>Clay<br>Collage/s<br>Comic/s OR Comics OR<br>Comicbook*<br>Drawing/s<br>Edutainment<br>Film/s<br>Filming<br>Fotonovela*<br>Photonovella*<br>Photo elicitation<br>Photo-led<br>Mandala/s<br>Mural<br>Paint/s<br>Painting<br>Photograph/s<br>Photovoice<br>Pictorial<br>Portrait/s<br>Potter<br>Sculpt*<br>Watercolour<br>Ceramics<br>Mosaic<br>Graffiti

		Sketch*<br>Colouring<br>Video*<br>Video ethnography<br>Movie<br>Cinema<br>visual narrative<br>Participatory video<br>Visual art<br>Visual storytelling<br>Graphic elicitation<br>Visual participatory research

Search strategy

OVID databases

S1: Youth OR Young OR Adolescen* OR teen* OR ((early OR young OR emerging) adj (adult))
S2: women OR woman OR female* OR girl*
S3: ((substance OR drug* OR alcohol OR opioid OR heroin OR cocaine OR cannabis OR marijuana OR nicotine OR tobacco OR stimulant OR hallucinogen) adj (addiction OR use OR abuse OR misuse OR dependen*)) OR ((substance OR drug* OR alcohol) adj1 (disorder))
S4: (art NOT (antiretroviral OR “anti retroviral” OR “HAART” OR “assisted reproductive therapy” OR “assisted reproductive therapies” OR “state of the art”)) OR arts OR artist OR artists OR artistic OR artistry OR artistries OR quilt* OR printmak* OR “print making” OR “print-making” OR “print maker” OR “print-maker” OR “print makers” OR “print-makers” OR “silk screen” OR “silk screens” OR “silk screened” OR “silk screening” OR "art-based" OR "arts-based" OR "creative effort" OR "creative efforts" OR "creative engagement" OR "creative expression" OR "creative expressions" OR "creative process" OR "creative processes" OR "creative medicine" OR "creative practice" OR "creative therapy" OR "creative therapies" OR "expressive activity" OR "expressive activities" OR animat* OR artwork* OR caricature* OR cartoon* OR clay OR collag* OR comic OR comics OR comicbook* OR drawing* OR edutainment OR film OR films OR filming OR fotonovela* OR photonovella* OR “photo elicitation” or “photo-elicitation” OR “photo-led” OR mandala* OR mural* OR paint* OR photograph* OR photovoice OR “photo voice” OR pictorial OR portrait* OR potter* OR sculpt* OR “water-color” OR “water-colors” OR “water-colour” OR “water-colours” OR “water color” OR “water colors” OR “water colour” OR “water colours” OR watercolour* OR watercolor* OR ceramics OR mosaic* OR graffiti OR aesthetic* OR esthetic* OR sketch* OR coloring OR colouring OR video* OR “video ethnography” OR movie* OR “motion picture" OR "motion pictures" OR cinema* OR “visual narrative” OR “participatory visual method” Or “participatory video” OR “visual art” OR “visual storytelling” OR “graphic elicitation” OR “visual participatory research” OR ((digital) adj (story OR stories OR storytelling OR narrat*))

EBSCO databases

S1: Youth OR Young OR Adolescen* OR teen* OR ((early OR young OR emerging) NEAR/0 (adult*))
S2: women OR woman OR female* OR girl*
S3: ((substance OR drug* OR alcohol OR opioid OR heroin OR cocaine OR cannabis OR marijuana OR nicotine OR tobacco OR stimulant OR hallucinogen) NEAR/0 (addiction OR use OR abuse OR misuse OR dependen*)) OR ((substance OR drug* OR alcohol) NEAR/1 (disorder))
S4: (art NOT (antiretroviral OR "anti retroviral" OR "HAART" OR "assisted reproductive therapy" OR "assisted reproductive therapies" OR "state of the art")) OR arts OR artist OR artists OR artistic OR artistry OR artistries OR quilt* OR printmak* OR "print making" OR "print-making" OR "print maker" OR "print-maker" OR "print makers" OR "print-makers" OR "silk screen" OR "silk screens" OR "silk screened" OR "silk screening" OR "art-based" OR "arts-based" OR "creative effort" OR "creative efforts" OR "creative engagement" OR "creative expression" OR "creative expressions" OR "creative process" OR "creative processes" OR "creative medicine" OR "creative practice" OR "creative therapy" OR "creative therapies" OR "expressive activity" OR "expressive activities" OR animat* OR artwork* OR caricature* OR cartoon* OR clay OR collag* OR comic OR comics OR comicbook* OR drawing* OR edutainment OR film OR films OR filming OR fotonovela* OR photonovella* OR "photo elicitation" or "photo-elicitation" OR "photo-led" OR mandala* OR mural* OR paint* OR photograph* OR photovoice OR "photo voice" OR pictorial OR portrait* OR potter* OR sculpt* OR "water-color" OR "water-colors" OR "water-colour" OR "water-colours" OR "water color" OR "water colors" OR "water colour" OR "water colours" OR watercolour* OR watercolor* OR ceramics OR mosaic* OR graffiti OR aesthetic* OR esthetic* OR sketch* OR coloring OR colouring OR video* OR "video ethnography" OR movie* OR "motion picture" OR "motion pictures" OR cinema* OR "visual narrative" OR "participatory visual method" Or "participatory video" OR "visual art" OR "visual storytelling" OR "graphic elicitation" OR "visual participatory research" OR ((digital) NEAR/0 (story OR stories OR storytelling OR narrat*))

Search date: 26th September 2024

OVID Medline
Search in title and abstracts
Filters: last 10 years, English

#	Search words	Hits
1	exp substance addiction/	82447
2	exp drug misuse/	8315
3	exp alcohol addiction/	13041
4	((substance OR drug* OR alcohol OR opioid OR heroin OR cocaine OR cannabis OR marijuana OR nicotine OR tobacco OR stimulant OR hallucinogen) adj (addiction OR abuse OR misuse OR dependen*)) OR ((substance OR drug* OR alcohol) adj1 (disorder))	60946
5	1 OR 2 OR 3 OR 4	111659
6	women OR woman OR female* OR girl*	3293904
7	Exp participatory research/	4207
8	((art) NOT (antiretroviral OR “anti retroviral” OR “HAART” OR “assisted reproductive therapy” OR “assisted reproductive therapies” OR “state of the art”)) OR arts OR artist OR artists OR artistic OR artistry OR artistries OR quilt* OR printmak* OR “print making” OR “print-making” OR “print maker” OR “print-maker” OR “print makers” OR “print-makers” OR “silk screen” OR “silk screens” OR “silk screened” OR “silk screening” OR "art-based" OR "arts-based" OR "creative effort" OR "creative efforts" OR "creative engagement" OR "creative expression" OR "creative expressions" OR "creative process" OR "creative processes" OR "creative medicine" OR "creative practice" OR "creative therapy" OR "creative therapies" OR "expressive activity" OR "expressive activities" OR animat* OR artwork* OR caricature* OR cartoon* OR clay OR collag* OR comic OR comics OR comicbook* OR drawing* OR edutainment OR film OR films OR filming OR fotonovela* OR photonovella* OR “photo elicitation” or “photo-elicitation” OR “photo-led” OR mandala* OR mural* OR paint* OR photograph* OR photovoice OR “photo voice” OR pictorial OR portrait* OR potter* OR sculpt* OR “water-color” OR “water-colors” OR “water-colour” OR “water-colours” OR “water color” OR “water colors” OR “water colour” OR “water colours” OR watercolour* OR watercolor* OR ceramics OR mosaic* OR graffiti OR aesthetic* OR esthetic* OR sketch* OR coloring OR colouring OR video* OR “video ethnography” OR movie* OR “motion picture" OR "motion pictures" OR cinema* OR “visual narrative” OR “participatory visual method” Or “participatory video” OR “visual art” OR “visual storytelling” OR “graphic elicitation” OR “visual participatory research” OR ((digital) adj (story OR stories OR storytelling OR narrat*))	567680
9	7 OR 8	571403
10	5 AND 6 AND 9	1197

Without MEDLINE in Embase and PsychInfo

OVID EMBASE
Search in title and abstracts
Filters: last 10 years, English

#	Search words	Hits
1	exp drug addiction/	49682
2	exp drug misuse/	1803
3	Exp alcohol abuse/	13301
4	exp alcohol addiction/	23871
5	((substance OR drug* OR alcohol OR opioid OR heroin OR cocaine OR cannabis OR marijuana OR nicotine OR tobacco OR stimulant OR hallucinogen) adj (addiction OR abuse OR misuse OR dependen*)) OR ((substance OR drug* OR alcohol) adj1 (disorder))	56177
6	1 OR 2 OR 3 OR 4 OR 5	78221
7	women OR woman OR female* OR girl*	3141654
8	((art) NOT (antiretroviral OR “anti retroviral” OR “HAART” OR “assisted reproductive therapy” OR “assisted reproductive therapies” OR “state of the art”)) OR arts OR artist OR artists OR artistic OR artistry OR artistries OR quilt* OR printmak* OR “print making” OR “print-making” OR “print maker” OR “print-maker” OR “print makers” OR “print-makers” OR “silk screen” OR “silk screens” OR “silk screened” OR “silk screening” OR "art-based" OR "arts-based" OR "creative effort" OR "creative efforts" OR "creative engagement" OR "creative expression" OR "creative expressions" OR "creative process" OR "creative processes" OR "creative medicine" OR "creative practice" OR "creative therapy" OR "creative therapies" OR "expressive activity" OR "expressive activities" OR animat* OR artwork* OR caricature* OR cartoon* OR clay OR collag* OR comic OR comics OR comicbook* OR drawing* OR edutainment OR film OR films OR filming OR fotonovela* OR photonovella* OR “photo elicitation” or “photo-elicitation” OR “photo-led” OR mandala* OR mural* OR paint* OR photograph* OR photovoice OR “photo voice” OR pictorial OR portrait* OR potter* OR sculpt* OR “water-color” OR “water-colors” OR “water-colour” OR “water-colours” OR “water color” OR “water colors” OR “water colour” OR “water colours” OR watercolour* OR watercolor* OR ceramics OR mosaic* OR graffiti OR aesthetic* OR esthetic* OR sketch* OR coloring OR colouring OR video* OR “video ethnography” OR movie* OR “motion picture" OR "motion pictures" OR cinema* OR “visual narrative” OR “participatory visual method” Or “participatory video” OR “visual art” OR “visual storytelling” OR “graphic elicitation” OR “visual participatory research” OR ((digital) adj (story OR stories OR storytelling OR narrat*))	313802
9	6 AND 7 AND 8	1182

OVID PsychInfo
Search in title and abstracts
Filters: last 10 years, English, includes peer-reviewed journals

#	Search words	Hits
1	exp drug addiction/	2203
2	exp drug abuse/	6227
3	Exp alcohol abuse/	5584
4	exp alcohol addiction/	7161
5	((substance OR drug* OR alcohol OR opioid OR heroin OR cocaine OR cannabis OR marijuana OR nicotine OR tobacco OR stimulant OR hallucinogen) adj (addiction OR abuse OR misuse OR dependen*)) OR ((substance OR drug* OR alcohol) adj1 (disorder))	20982
6	1 OR 2 OR 3 OR 4 OR 5	24601
7	women OR woman OR female* OR girl*	194465
8	Exp participatory action research/	1764
9	((art) NOT (antiretroviral OR “anti retroviral” OR “HAART” OR “assisted reproductive therapy” OR “assisted reproductive therapies” OR “state of the art”)) OR arts OR artist OR artists OR artistic OR artistry OR artistries OR quilt* OR printmak* OR “print making” OR “print-making” OR “print maker” OR “print-maker” OR “print makers” OR “print-makers” OR “silk screen” OR “silk screens” OR “silk screened” OR “silk screening” OR "art-based" OR "arts-based" OR "creative effort" OR "creative efforts" OR "creative engagement" OR "creative expression" OR "creative expressions" OR "creative process" OR "creative processes" OR "creative medicine" OR "creative practice" OR "creative therapy" OR "creative therapies" OR "expressive activity" OR "expressive activities" OR animat* OR artwork* OR caricature* OR cartoon* OR clay OR collag* OR comic OR comics OR comicbook* OR drawing* OR edutainment OR film OR films OR filming OR fotonovela* OR photonovella* OR “photo elicitation” or “photo-elicitation” OR “photo-led” OR mandala* OR mural* OR paint* OR photograph* OR photovoice OR “photo voice” OR pictorial OR portrait* OR potter* OR sculpt* OR “water-color” OR “water-colors” OR “water-colour” OR “water-colours” OR “water color” OR “water colors” OR “water colour” OR “water colours” OR watercolour* OR watercolor* OR ceramics OR mosaic* OR graffiti OR aesthetic* OR esthetic* OR sketch* OR coloring OR colouring OR video* OR “video ethnography” OR movie* OR “motion picture" OR "motion pictures" OR cinema* OR “visual narrative” OR “participatory visual method” Or “participatory video” OR “visual art” OR “visual storytelling” OR “graphic elicitation” OR “visual participatory research” OR ((digital) adj (story OR stories OR storytelling OR narrat*))	61145
10	10 OR 11	62579
11	6 AND 7 AND 10	207

SCOPUS
Search in title, abstracts and keywords
Filters: 2014 to present, English, article, review, conference paper

S1: (women OR woman OR female* OR girl*)

S2: ((substance OR drug* OR alcohol OR opioid OR heroin OR cocaine OR cannabis OR marijuana OR nicotine OR tobacco OR stimulant OR hallucinogen) Pre/0 (addiction OR abuse OR misuse OR dependen*)) OR ((substance OR drug* OR alcohol) Pre/0 (disorder))

S3: ((art) AND NOT (antiretroviral OR “anti retroviral” OR “HAART” OR “assisted reproductive therapy” OR “assisted reproductive therapies” OR “state of the art”)) OR arts OR artist OR artists OR artistic OR artistry OR artistries OR quilt* OR printmak* OR “print making” OR “print-making” OR “print maker” OR “print-maker” OR “print makers” OR “print-makers” OR “silk screen” OR “silk screens” OR “silk screened” OR “silk screening” OR "art-based" OR "arts-based" OR "creative effort" OR "creative efforts" OR "creative engagement" OR "creative expression" OR "creative expressions" OR "creative process" OR "creative processes" OR "creative medicine" OR "creative practice" OR "creative therapy" OR "creative therapies" OR "expressive activity" OR "expressive activities" OR animat* OR artwork* OR caricature* OR cartoon* OR clay OR collag* OR comic OR comics OR comicbook* OR drawing* OR edutainment OR film OR films OR filming OR fotonovela* OR photonovella* OR “photo elicitation” or “photo-elicitation” OR “photo-led” OR mandala* OR mural* OR paint* OR photograph* OR photovoice OR “photo voice” OR pictorial OR portrait* OR potter* OR sculpt* OR “water-color” OR “water-colors” OR “water-colour” OR “water-colours” OR “water color” OR “water colors” OR “water colour” OR “water colours” OR watercolour* OR watercolor* OR ceramics OR mosaic* OR graffiti OR aesthetic* OR esthetic* OR sketch* OR coloring OR colouring OR video* OR “video ethnography” OR movie* OR “motion picture” OR "motion pictures" OR cinema* OR “visual narrative” OR “participatory visual method” Or “participatory video” OR “visual art” OR “visual storytelling” OR “graphic elicitation” OR “visual participatory research” OR ((digital) Pre/0 (story OR stories OR storytelling OR narrat*))

S4: S1 AND S2 AND S3= 1950

Web of Science
Search in title, abstracts and keywords
Filters: 1st August 2014 to 26th September 2024, English, article, review

S1: (women OR woman OR female* OR girl*)
S2: (((substance OR drug* OR alcohol OR opioid OR heroin OR cocaine OR cannabis OR marijuana OR nicotine OR tobacco OR stimulant OR hallucinogen) NEAR/0 (addiction OR use OR abuse OR misuse OR dependen*)) OR ((substance OR drug* OR alcohol) NEAR/1 (disorder)))
S3: (art NOT (antiretroviral OR “anti retroviral” OR “HAART” OR “assisted reproductive therapy” OR “assisted reproductive therapies” OR “state of the art”)) OR arts OR artist OR artists OR artistic OR artistry OR artistries OR quilt* OR printmak* OR “print making” OR “print-making” OR “print maker” OR “print-maker” OR “print makers” OR “print-makers” OR “silk screen” OR “silk screens” OR “silk screened” OR “silk screening” OR "art-based" OR "arts-based" OR "creative effort" OR "creative efforts" OR "creative engagement" OR "creative expression" OR "creative expressions" OR "creative process" OR "creative

processes" OR "creative medicine" OR "creative practice" OR "creative therapy" OR "creative therapies" OR "expressive activity" OR "expressive activities" OR animat* OR artwork* OR caricature* OR cartoon* OR clay OR collag* OR comic OR comics OR comicbook* OR drawing* OR edutainment OR film OR films OR filming OR fotonovela* OR photonovella* OR “photo elicitation” or “photo-elicitation” OR “photo-led” OR mandala* OR mural* OR paint* OR photograph* OR photovoice OR “photo voice” OR pictorial OR portrait* OR potter* OR sculpt* OR “water-color” OR “water-colors” OR “water-colour” OR “water-colours” OR “water color” OR “water colors” OR “water colour” OR “water colours” OR watercolour* OR watercolor* OR ceramics OR mosaic* OR graffiti OR aesthetic* OR esthetic* OR sketch* OR coloring OR colouring OR video* OR “video ethnography” OR movie* OR “motion picture" OR "motion pictures" OR cinema* OR “visual narrative” OR “participatory visual method” Or “participatory video” OR “visual art” OR “visual storytelling” OR “graphic elicitation” OR “visual participatory research” OR ((digital) NEAR/0 (story OR stories OR storytelling OR narrat*))

S4: S1 AND S2 AND S3= 1240
